# New Pathological and Clinical Insights in Endometrial Cancer in View of the Updated ESGO/ESTRO/ESP Guidelines

**DOI:** 10.3390/cancers13112623

**Published:** 2021-05-26

**Authors:** Angela Santoro, Giuseppe Angelico, Antonio Travaglino, Frediano Inzani, Damiano Arciuolo, Michele Valente, Nicoletta D’Alessandris, Giulia Scaglione, Vincenzo Fiorentino, Antonio Raffone, Gian Franco Zannoni

**Affiliations:** 1 Unità di Ginecopatologia e Patologia Mammaria, Dipartimento Scienze della Salute della Donna, del Bambino e di Sanità Pubblica, Fondazione Policlinico Universitario A. Gemelli IRCCS, Largo A. Gemelli 8, 00168 Roma, Italy; angela.santoro@policlinicogemelli.it (A.S.); giuangel86@hotmail.it (G.A.); antonio.travaglino.ap@gmail.com (A.T.); frediano.inzani@policlinicogemelli.it (F.I.); damiano.arciuolo@policlinicogemelli.it (D.A.); dr.valente.m@gmail.com (M.V.); ndalessandris@gmail.com (N.D.); scaglione.giulia90@gmail.com (G.S.); vincenzof.89@hotmail.it (V.F.); 2 Gynecology and Obstetrics Unit, Department of Neuroscience, Reproductive Sciences and Dentistry, School of Medicine, University of Naples Federico II, 80131 Naples, Italy; anton.raffone@gmail.com; 3 Istituto di Anatomia Patologica, Università Cattolica del Sacro Cuore, Largo A. Gemelli 8, 00168 Roma, Italy

**Keywords:** endometrial carcinoma, TCGA, serous carcinoma, clear cell carcinoma, undifferentiated carcinoma, CTNNB1, prognosis

## Abstract

**Simple Summary:**

Histopathological classification of endometrial carcinoma has evidenced two main groups with different biological behavior: low-grade (G1–G2) and high-grade (G3) endometrial tumors. Moreover, the Cancer Genome Atlas (TCGA) documented four molecular categories with distinct clinical, pathologic, and molecular features: POLE/ultramutated (7% of cases) microsatellite instability (MSI)/hypermutated (28%), copy-number low/endometrioid (39%), and copy-number high/serous-like (26%). The aim of the present paper is to review all endometrial carcinoma histotypes in light of the morphological and molecular prognostic TCGA groups.

**Abstract:**

Endometrial carcinoma represents the most common gynecological cancer in Europe and the USA. Histopathological classification based on tumor morphology and tumor grade has played a crucial role in the management of endometrial carcinoma, allowing a prognostic stratification into distinct risk categories, and guiding surgical and adjuvant therapy. In 2013, The Cancer Genome Atlas (TCGA) Research Network reported a large scale molecular analysis of 373 endometrial carcinomas which demonstrated four categories with distinct clinical, pathologic, and molecular features: POLE/ultramutated (7% of cases) microsatellite instability (MSI)/hypermutated (28%), copy-number low/endometrioid (39%), and copy-number high/serous-like (26%). In the present article, we report a detailed histological and molecular review of all endometrial carcinoma histotypes in light of the current ESGO/ESTRO/ESP guidelines. In particular, we focus on the distribution and prognostic value of the TCGA groups in each histotype.

## 1. Introduction

Endometrial carcinoma is the sixth most commonly diagnosed cancer and the 14th leading cause of cancer death in women worldwide [1]. It represents the most common gynecological cancer in Europe and the USA [2,3]. Historically, the first classification of endometrial carcinoma, as proposed by Bokhman in 1983, recognized Type I (endometrioid-type) and Type II (serous-type) endometrial cancers based on clinical and endocrine features. Type I carcinomas occur in obese women with hyperlipidemia and signs of hyperestrogenism and are characterized by a low grade (G1–2), early stage at presentation, sensitivity to progestins, and good prognosis. Type II carcinomas occur in women with no signs of hyperestrogenism and are characterized by high grade (G3), higher stage at presentation, decreased sensitivity to progestins, and poor prognosis [4]. However, the Bokhman classification could not account for the high morphological and molecular heterogeneity of endometrial carcinoma, and the correct place of some histotypes in such classification has never been defined [5,6,7]. Histopathological classification based on tumor morphology and tumor grade has played a crucial role in the management of endometrial carcinoma, allowing a prognostic stratification into distinct risk categories, and guiding surgical and adjuvant therapy. Low-grade (G1–2) endometrial endometrioid carcinomas (EEC) have been regarded as the most prognostically favorable subset of endometrial carcinoma. Non-endometrioid carcinomas, which are all graded G3 and mainly include serous endometrial carcinoma (SEC) and clear cell endometrial carcinoma (CCEC), have been considered high-risk histotypes; G3 EEC has been considered prognostically intermediate between the former and the latter [8]. Undifferentiated/dedifferentiated endometrial carcinoma (UEC/DEC) and uterine carcinosarcoma (UCS), recently classified as variants of endometrial carcinoma, have also been included in the non-endometrioid group [8,9]. Less common histotypes include neuroendocrine endometrial carcinoma (NEEC), mesonephric-like endometrial carcinoma (MLEC), and gastric/gastrointestinal-type endometrial carcinoma (GTEC) [9]. Other relevant histopathological prognostic factors, including deep myometrial invasion and lymphovascular space invasion (LVSI), have been used to substratif y the risk, especially in EEC [8]. Unfortunately, the pathologic evaluation of prognostic factors is beset by challenges, including the reproducibility of histologic classification and International Federation of Gynecology and Obstetrics (FIGO) grading. There is frequently an overlap between histologic subtypes and grade determination complicating clinical decision making. Therefore, interobserver diagnostic agreement is still suboptimal, particularly among the high-grade histotypes and in frozen section specimens [10,11]. In 2013, The Cancer Genome Atlas (TCGA) Research Network reported a large scale, integrated genomic, transcriptomic, and proteomic analysis of 373 endometrial carcinomas, including 307 EECs, 53 SECs, and 13 mixed cases. The study performed whole exome sequence analysis, transcriptome sequence analysis, genomic copy number analysis, protein array analysis, microsatellite stability testing, and methylation profiling. Four categories of ECs with distinct clinical, pathologic, and molecular features were identified: POLE/ultramutated (7% of cases) microsatellite instability (MSI)/hypermutated (28%), copy-number low/endometrioid (39%), and copy-number high/serous-like (26%). The POLE/ultramutated group is characterized by somatic mutations in the exonuclease domain of POLE, which encodes the catalytic subunit of DNA polymerase epsilon involved in nuclear DNA replication and repair. As their name suggests, ultramutated tumors have an extraordinarily high mutation rate (232 × 10^−6^ mutations per megabase). The POLE/ultramutated group included both low-grade and high-grade EECs and showed excellent prognosis with no recurrence regardless of the FIGO grade. The MSI/hypermutated group is characterized by MSI, mostly caused by MLH1 promoter methylation, and a high mutational rate (18 × 10^−6^ mutations per megabase). The MSI/hypermutated group included both low-grade and high-grade EEC (similarly to the POLE/ultramutated group) and is characterized by intermediate prognosis. The copy-number low/endometrioid group is characterized by low mutational rate (2.9 × 10^−6^ mutations per megabase), with no MSI or POLE mutations and with a low degree of somatic copy-number alteration (SCNA). The copy-number low/endometrioid group mainly includes low-grade EEC and has been compared to the prototypical Bokhman type I category of endometrial carcinoma; the overall prognosis in this group is intermediate. The copy-number high/serous-like group is characterized by low mutational rate (2.3 × 10^−6^ mutation per megabase) but extensive SCNA, with TP53 mutation in 90% of cases. The copy-number high/serous like group mainly includes SECs and has been compared to the prototypical Bokhman type II category; this group shows poor prognosis [12]. The outstanding prognostic value of the TCGA molecular classification has been confirmed in subsequent studies [13]. Other research groups, in particular the Leiden/PORTEC group and the Vancouver/ProMisE group, have helped to improve the clinical applicability of the TCGA classification by finding cheaper surrogates of molecular prognostic markers. In detail, immunohistochemical assessment of mismatch repair (MMR) proteins and p53 has been used as a surrogate of molecular testing of MSI and SCNA, respectively; a reliable surrogate of POLE sequencing has not yet been identified. Such surrogate classification defines four groups which reflect the TCGA prognostic groups: POLE-mutated (POLEmut, which is the same as the POLE/ultramutated group), MMR-deficient (MMRd, surrogate of the MSI/hypermutated group), p53-abnormal (p53abn, surrogate of the copy-number high/serous-like group), and no specific molecular profile (NSMP, surrogate of the copy-number low/endometrioid group) [14,15,16,17].

**Figure 1:**
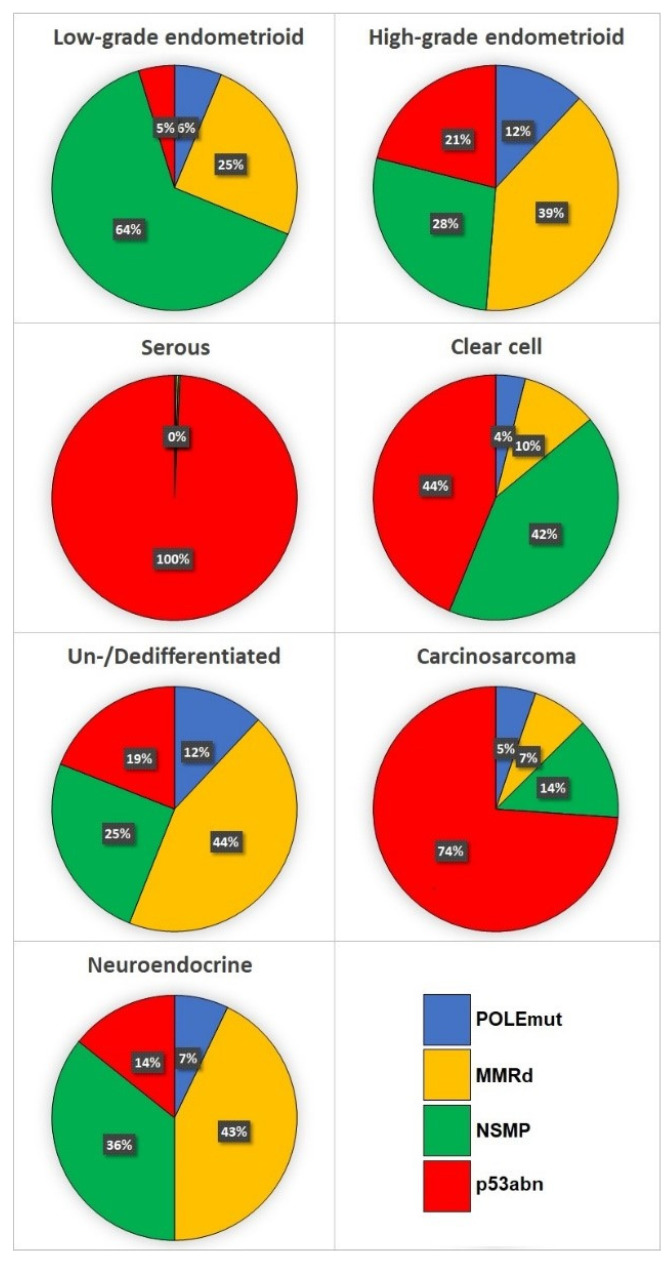
Distribution of TCGA molecular groups according to endometrial carcinoma histotype.

**Figure 2:**
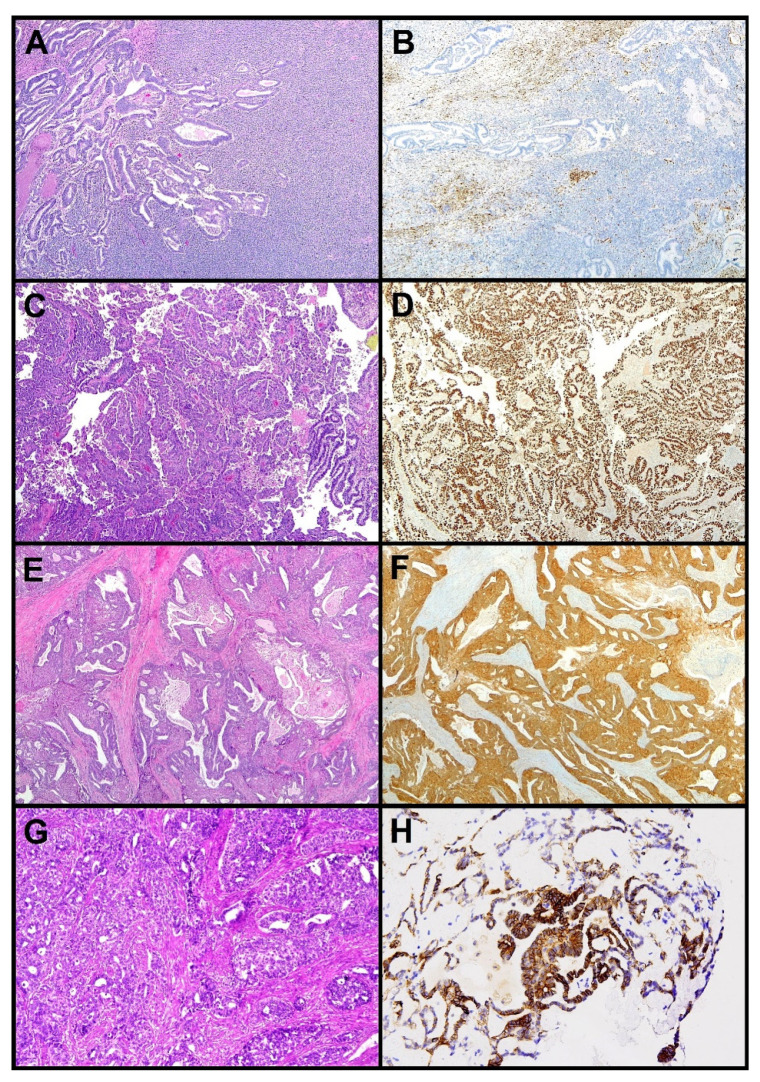
Molecular landscape of endometrial carcinoma: the role of the immunohistochemical surrogates. (**A**,**B**) Dedifferentiated carcinoma with mismatch repair deficiency. (**C**,**D**) Serous carcinoma with abnormal p53 expression. (**E**,**F**) Endometrioid carcinoma with nuclear beta-catenin accumulation. (**G**,**H**) Endometrioid carcinoma with positivity for L1CAM in >10% of tumor cells (H&E, LSAB, 10×).

Distribution of endometrial carcinoma histotypes according to TCGA molecular groups, is illustrated in Figure 1. Figure 2 illustrates part of the molecular landscape of endometrial carcinoma, highlighting the role of the immunohistochemical surrogates.

In 2020, the European Society of Gynaecological Oncology (ESGO), the European Society for Radiotherapy and Oncology (ESTRO), and the European Society of Pathology (ESP) published their joint guidelines for the management of endometrial carcinoma, for the first time incorporating the TCGA findings in the risk stratification of endometrial carcinoma. The ESGO/ESTRO/ESP guidelines propose two alternative approaches to stratify the risk in endometrial carcinoma, based on whether molecular classification is available. The two approaches are detailed in Table 1. The most evident novelties are that all POLEmut carcinomas up to FIGO stage II, regardless of FIGO grade, histotype, or LVSI, are included in the low-risk group; this implies that these cases would be managed by observation alone, with no need for adjuvant treatment. On the other hand, p53mut EECs are lumped together with non-endometrioid carcinomas in the high-risk group; in the absence of myometrial invasion, these tumors are considered at intermediate risk [18]. However, there are still several points that should be clarified, since prognostic value of the TCGA molecular groups might vary across the different histotypes of endometrial carcinoma [19]. Furthermore, other clinicopathological and molecular factors might have an independent prognostic value in the context of the TCGA classification [13]. This has highlighted the need for a more integrated approach to the prognostic stratification of endometrial carcinoma, which is a major goal of the scientific research in this field. In this review, we deal with the several endometrial carcinoma histotypes in light of the current ESGO/ESTRO/ESP guidelines. In particular, we focus on the distribution and prognostic value of the TCGA groups in each histotype, proposing refinements in the molecular-based risk stratification and discussing the possible tailored therapeutic approaches. We also discuss the issue related to the routine application of the molecular classifier in the common practice.

## 2. Endometrioid Carcinoma (EEC)

EEC is the most common histotype of endometrial carcinoma. EEC often arises from atypical endometrial hyperplasia/endometrioid intraepithelial neoplasia (AEH/EIN), which is its recognized precancerous lesion. EEC is characterized by glandular structures lined by columnar/cuboidal cells with round/ovoidal pseudostratified nuclei and a smooth luminal surface; nuclear atypia is most commonly low-grade. Altered differentiations, such as mucinous, squamous, and morular, are common and are used as confirmatory features of endometrioid histotype. Pathological grading is crucial for the risk stratification and management of endometrioid carcinoma. EECs are subdivided into “low-grade” (FIGO grade 1–2) and “high-grade” (FIGO grade 3), based on the percentage of solid growth pattern (< or ≥50%); one grade is added in the case of marked nuclear atypia [9,20]. Low-grade EEC has been defined as the prototypical Bokhman type I carcinoma and has been associated with good prognosis [4]. On the other hand, the place of high-grade EEC in the Bokhman classification has been debated, since it shows features of both type I and type II carcinoma [6]. According to the TCGA classification, most low-grade EEC (>60%) fall into the NSMP group; about ¼th fall into the MMRd group, while the POLEmut and p53abn groups are uncommon. Instead, high-grade EECs show a significantly higher proportion in the MMRd group (which is the most represented), p53mut group, and POLEmut group [21]. The ESGO/ESTRO/ESP guidelines consider all POLEmut EEC up to stage II at low risk regardless of other pathological factors [18], indicating that these cases do not need adjuvant treatment. Indeed, a recent meta-analysis showed a 0% risk of nodal metastases in POLEmut carcinomas [22]. The biological behavior of POLEmut EEC at FIGO stage >II is still unclear, given the rarity of these cases [18]. All p53abn EECs lumped together with non-endometrioid carcinomas; hence, they are considered at intermediate risk in the absence of myometrial invasion and at high-risk in the case of myoinvasive disease. Such classification is based on the poor prognosis of the p53abn group, which is significantly worse than that of the other TCGA groups [12]. It is unclear whether p53abn EEC and serous carcinoma have the same prognosis, since the results in this regard appear conflicting [23,24]. However, given the possibility of mixed EEC and serous carcinoma and the possible difficulties in differentiating between p53abn EEC and serous carcinoma, considering a different management for these two entities might not be appropriate [20,25]. For the MMRd and NSMP groups, which have intermediate prognosis, FIGO grade, LVSI, and depth of myometrial invasion are crucial factors for the ESGO/ESTRO/ESP risk stratification. Indeed, MMRd/NSMP EEC are considered at low risk in the case of low FIGO grade, stage IA (<50% myometrial invasion), and no or focal LVSI. Stage IA G3 EEC and stage IB (≥50% myometrial invasion) G1–2 EEC are considered at intermediate risk, while FIGO IB G3 EEC and/or the presence of substantial (>1 focus) LVSI require a high-intermediate risk classification [12]. The role of LVSI is supported by its independent prognostic value and the reproducibility of its assessment [17,26]. In the MMRd group, both LVSI and deep myometrial invasion were found as independent prognostic factors, while a high FIGO grade was not [27]. It appears, therefore, necessary to further assess whether the prognosis of MMRd EC varies between low-grade and high-grade EEC, especially considering that POLEmut carcinomas do not seem to be prognostically affected by the FIGO grade [12]. In addition, MMRd EECs with MLH1 promoter methylation seem to have a worse prognosis than MMRd EEC with the mutation of MMR genes [27], offering a possible substratification of the MMRd group. Regarding the NSMP group, the absence of specific molecular signatures seems to be accompanied by a more heterogeneous biological behavior compared to the other TCGA groups. The Leiden/PORTEC group identified CTNNB1 exon 3 mutations and L1CAM expression as further independent prognostic factors in NSMP EECs. In particular, the presence of CTNNB1 mutation may identify a subset at intermediate prognosis (similar to that of the MMRd group), while an expression of L1CAM in >10% of cancer cells may indicate poor prognosis (similar to that of the p53abn group); NSMP EECs that do not show these factors may have a good prognosis, similar to that of the POLEmut group [17]. The reliability of such refined risk stratification system is currently being assessed in a prospective trial [28]. Such substratification would allow overcoming the heterogeneous prognosis of the NSMP group and leading to a more precise risk stratification. However, the trans-PORTEC initiative only involved high-intermediate risk EEC, and thus, we cannot draw conclusions about the generalizability of these results [17].

There are other morphologic factors that might have an independent prognostic value in EEC, such as a tumor budding, microcystic, elongated, and fragmented (MELF) pattern of invasion and WT1 immunohistochemical expression [29,30,31], although data in this regard are scarce. The prognostic significance of these factors, their reproducibility and their possible integration in the current risk stratification system need to be further investigated.

## 3. Serous Carcinoma (SEC)

SEC is the prototypical Bokhman type II carcinoma, i.e., mostly arises in postmenopausal women, is not associated with estrogens, and shows poor prognosis [4,9]. A proposed model for SEC carcinogenesis starts with TP53 mutation in resting endometrium and evolves towards serous intraepithelial carcinoma through a precancerous phase defined “endometrial glandular dysplasia” [32]. SEC may show papillary, glandular, or solid growth patterns. Distinctive features of SEC are a scalloped epithelial surface with exfoliation of tumoral cells, a lack of polarization with striking nuclear atypia, and a high mitotic index [9,20]. SEC have shown a quite homogeneous molecular background characterized by TP53 mutations, which can be detected by p53 immunohistochemistry and is useful for differential diagnosis in difficult cases [4,20,25]. Consistently, SECs almost invariably fall into the p53abn TCGA group, which has indeed been termed the “serous-like” group [12,33]. Similarly to the other non-endometrioid histotypes, SEC is placed in the high-risk category in the case of myoinvasive disease and in the intermediate-risk category when there is an absence of myometrial invasion [18]. Although SEC appears as the most prognostically and molecularly homogeneous histotype of endometrial carcinoma, there are some exceptions that should be remarked. First of all, POLEmut EEC may show serous-like morphological features with striking nuclear atypia [20,34]. Furthermore, mixed EEC and SEC not rarely show POLEmut or MMRd signature, especially women <60 years. In these cases, a p53 abnormal expression pattern (reflecting TP53 mutations) may occur as a consequence of the high mutational rate, but it does not affect prognosis [35]. Therefore, even in the presence of morphologically unequivocal SC, applying the TCGA classification appears crucial to avoid severe overtreatment of patients. POLEmut and MMRd cases with serous features indeed show a prognosis comparable to that of their EEC counterpart, supporting the need for a similar management [34,35].

## 4. Clear Cell Carcinoma (CCEC)

CCEC has traditionally been lumped together SEC in the Bokhman type II category [36]. CCEC is characterized by cuboidal/polygonal cells with clear or eosinophilic cytoplasm and “hobnail” appearance, arranged in tubulo-cystic, papillary, or solid structures. The presence of clear cells areas may not rarely be observed in EEC and SEC [4,20]. To date, no univocal precursors of CCEC have been identified, although putative precancerous lesions with heterogeneous morphology have been described [37]. The typical immunophenotype of CCEC is characterized by positivity for Napsin-A, HNF-1β, and AMACR and negativity for estrogen and progesterone receptors [4,38]. CCEC has been shown to be a molecularly heterogeneous entity, which share genomic alterations with both EEC and SEC [39]. According to the TCGA classification, almost half of CCEC fall into the p53abn group, consistently with the overall poor prognosis of this histotype. A significant proportion (about 40%) falls into the NSMP group. POLEmut CCEC are rare, while the percentage of MMRd CCEC has been shown to vary among different studies [40,41]. Interestingly, the MMRd signature have been commonly described in mixed EEC and CCEC [42]. The ESGO/ESTRO/ESP guidelines lump CCEC together with the other non-endometrioid histotypes. However, while the prognosis of p53abn CCEC is considered poor, the prognosis of MMRd and NSMP CCEC is less defined. Such a knowledge gap is clearly highlighted in the ESGO/ESTRO/ESP guidelines [18]. Interestingly, the few studied focused on CCEC and mixed EEC/CCEC showed excellent prognosis for MMRd CCEC [42,43,44]. Although the prognosis of the MMRd and POLEmut groups in these studies appeared similar [43,44], it is not reasonable to conclude that MMRd CCEC deserves a “low-risk” classification. In fact, MMRd EEC are characterized by an intermediate prognosis [12], and it appears unlikely that CCEC of the same group have better prognosis. Instead, it seems more reasonable to lump MMRd CCEC together with MMRd EEC. Regarding NSMP CCEC, they showed worse prognosis than MMRd CCEC but better prognosis than p53abn CCEC [43,44]. It has been suggested that NSMP CCCs behave more aggressively than G3 EECs of the same group [43]. In the absence of further evidence, it appears appropriate to manage NSMP CCECs in the same way as p53abn CCECs. POLEmut CCECs are considered at low risk, similar to all other POLEmut endometrial carcinomas, regardless of the histotype [18].

## 5. Mixed Carcinoma

The term “mixed carcinoma” indicates the presence of two different endometrial carcinoma component (one of which is SEC or CCEC), with the minor component accounting for at least 5% of the tumoral area; each component typically shows an immunophenotype that can be superimposed on the correspondent pure histotype. Given the presence of an SEC or CCEC component, mixed carcinomas are considered of high grade by definition [4,20], and are lumped together with non-endometrioid carcinomas in the ESGO/ESTRO/ESP guidelines [18]. However, the prognosis of mixed carcinomas may be highly heterogeneous and appears strongly affected by the TCGA group. Previous studies showed a significant proportion of MMRd and POLEmut signatures in mixed carcinomas containing an EEC component, especially in younger women; such signatures are associated with good prognosis [35,42,45]. On this account, it might be appropriate to consider MMRd mixed carcinomas as analogous to MMRd EEC in terms of risk stratification. As discussed for pure histotype, all POLEmut mixed carcinomas are already considered to be at low risk by the ESGO/ESTRO/ESP guidelines [18]. In the absence of POLEmut and MMRd signatures, it remains appropriate to consider all mixed carcinomas analogously to SEC, given the overall unfavorable prognosis of these tumors [46]. A point of concern may be the possibility of different TCGA signatures in the different components of a mixed carcinoma. Matrai et al. reported the presence of POLE mutation limited to the SEC component of a mixed EEC/SEC [47]. In these cases, the prognosis might be driven by the component with the worse molecular signature. This raises the concern that a POLE mutation detected by sequencing might not be shared by all neoplastic clones. However, given the consistently excellent prognosis demonstrated by POLEmut carcinomas, we may reasonably hypothesize that such a condition is very rare. In fact, van Esterik et al. suggested that intratumoral heterogeneity had a limited impact on molecular risk assessment [48]. The possibility of a POLE mutation confined to a single component might deserve further attention.

## 6. Undifferentiated/Dedifferentiated Carcinoma (UEC/DEC)

UEC/DEC is a recently described variant of endometrial carcinoma. According to the WHO classification of gynecological tumors, UEC is characterized by sheets of medium-sized, monotonous discohesive cells with absence of any obvious glandular, nested, or trabecular architecture. UEC is often associated with a low-grade EEC component, configurating the so-called “dedifferentiated carcinoma” (DEC) [4,20]. Recent studies provided new insights on the morphological heterogeneity of UEC/DEC. In fact, UEC may show epithelioid, spindled, rhabdoid, or bizarre giant cells, with sometimes myxoid stroma [49]. The differentiated component of DEC may be high-grade EEC or even SEC [50]. UEC/DEC might be confused with high-grade EEC, SEC with solid growth pattern, NEEC, carcinosarcoma, or sarcomas, and is probably underrecognized in the common practice [51]. Most UEC/DEC show a loss of SWI/SNF complex proteins expression, which seem to be associated with dedifferentiation [52]. Consistently regarded as an aggressive entity, UEC/DEC has shown molecular heterogeneity. All four TCGA groups are represented in UEC/DEC, with a distribution similar to that of high-grade EEC (consistently with the “endometrioid” lineage of most UEC/DEC) [21,53]. The MMRd group is the most represented (about half of cases) [53]. Consistently with what observed in the other histotypes, POLEmut UEC/DEC have shown excellent prognosis, deserving the inclusion in the low-grade risk category of the ESGO/ESTRO/ESP system [18,52]. Significant prognostic differences could not be found among the three other groups, which showed poor prognosis. However, it has recently emerged that the loss of expression of certain SWI/SNF complex proteins expression, i.e., ARID1B, SMARCA4, and SMARCB1, is associated with very poor prognosis, even worse than that of carcinosarcomas [49,52]. We may hypothesize that these SWI/SNF-deficient UEC/DEC might deserve a still more aggressive management than non-endometroid carcinomas and be considered at high risk even in the absence of myometrial invasion, similarly to the recommendation of the NCCN (National Comprehensive Cancer Network) guidelines [54]. The prognosis of POLE-wild-type, SWI/SNF-proficient UEC/DEC remains to be defined; in such selected subsets, it is possible that MMRd cases may have improved prognosis, as discussed for other non-endometrioid histotypes. In this regard, a recent study suggested that MMRd carcinosarcomas (which often have a UEC/DEC component) may have improved prognosis compared to conventional carcinosarcomas [55]. Based on what observed in other histotypes, the prognosis of p53abn cases is expected to be poor, while the prognosis of NSMP cases is difficult to predict. In the absence of further evidence, it appears appropriate to continue considering all POLE-wild-type, SWI/SNF-proficient UEC/DEC in the non-endometrioid group together with SEC and p53abn EEC.

## 7. Uterine Carcinosarcoma (UCS)

UCS is an aggressive biphasic neoplasm composed by a carcinomatous and a sarcomatous component [4]. Previously classified among uterine sarcomas or among mixed Müllerian tumors [56,57], UCS is now listed among endometrial carcinoma histotypes [4,20]. Indeed, it is now accepted that UCS has epithelial derivation and that the sarcomatous component develops secondarily. The carcinomatous component of UCS is typically high grade and is most commonly SEC, although any histotype can be found. The sarcomatous component is also of high grade and may be homologous or heterologous, the latter defined by the presence of differentiation towards extrauterine mesenchymal tissues (e.g., rhabdomyosarcoma, chondrosarcoma, osteosarcoma, and liposarcoma) [4,20,58]. By adopting the TCGA molecular classification, the vast majority (>70%) of UCSs fall into the p53abn group, consistently with the frequent serous lineage and the poor prognosis [59]. The ESGO/ESTRO/ESP guidelines place UCS in the non-endometrioid group together with SEC; CCEC and UEC/DEC [18]. However, there is evidence supporting that p53abn UCS might be more aggressive than SEC [60,61,62]. In this regard, the NCCN guidelines recommend adjuvant treatment for UCS even in the case of no myometrial invasion and no residual tumor on hysterectomy specimen, while this recommendation is not made for SC and CCEC [54]. The need for a more aggressive approach in UCS compared to SC deserves further investigation. The NSMP group accounts for 10–15% of cases and seems to have a prognosis similar to that of the p53abn group. The rare POLEmut UCS have shown excellent prognosis, such as POLEmut carcinomas of other histotypes [59,63,64]. The MMRd group have shown the most variable prevalence among the TGCA groups in UCS. Indeed, the proportion of MMRd UCS varied from 3% to over 40% [63,65]; in our previous meta-analysis, the pooled estimated prevalence was 7.3% [59]. Similarly, the prognostic significance of the MMRd group in UCS was not consistent. Recent evidence suggests that the MMRd signature in UCS is associated improved prognosis [55]; however, other authors reported similar outcomes between MMRd and other groups. Remarkably, the study with the higher prevalence of the MMRd group also showed the worst prognosis for this group [65]. We might hypothesize that the high prevalence and poor prognosis of the MMRd group is due to the inclusion of highly aggressive UDC/DEC. In fact, DEC may mimic UCS, shows a MMRd signature in over 40% of cases, and may have very poor prognosis in the case of SWI/SNF-deficiency (as discussed above) [52,53]. On this account, we believe that an accurate prognostic evaluation of MMRd UCS should take place after careful exclusion of UEC/DEC cases and especially of SWI/SNF cases. We believe that MMRd UCS may really have improved prognosis, presumably similar to that of MMRd carcinomas of other histotypes. Further studies are necessary in this field.

## 8. Neuroendocrine Carcinoma (NEEC)

NEEC is a rare histotype of endometrial carcinoma, accounting for less than 1% of all cases. NEEC is characterized by the expression of at least one neuroendocrine marker (chromogranin, synaptophysin, and CD56) in at least 10% of tumoral cells; however, this feature is not specific and needs to be correlated with histomorphology. NEEC can be subdivided into small cell NEEC, characterized by hyperchromatic nuclei with high nuclear-to-cytoplasmic ratio, molding, and crushing artifacts, and large cell NEEC, characterized by appreciable cytoplasm, coarse nuclear chromatin with prominent nucleoli, and nested growth with peripheral nuclear palisading. Endometrial NEEC is often found admixed with other components, especially EEC [66]. The prognosis of gynecological NEECs is described as poor [67]. Given its rarity, the molecular background of NEEC is less studied than other histotypes. A recent study showed that all 4 TCGA groups were represented in a cohort of 14 NEECs (4 pure and 10 admixed with a second component) [66]. The most represented was the MMRd group, which accounted for more than 40% of cases; this percentage was consistent with previously published data [66,68]. Interestingly, all MMRd NEECs were mixed EEC/NEEC (except for one case that was UCS with a NEEC component). Out of five MMRd NEC cases with known follow-up, four were alive with no evidence of disease and one died of disease. Only one NEC case was POLEmut; this was a mixed EEC/NEEC case with unknown follow-up. The p53abn group included two pure small cell NEECs; one of these patients had a known follow-up and died of disease. NSMP NEEC included both pure large cell NEEC and mixed EEC/large cell NEEC; half of these patients died of disease [66]. As discussed for other non-endometrioid histotypes, it seems that POLEmut and MMRd NEEC mostly included cases (either small or large cell) admixed with an EEC component; these cases might be prognostically similar to their EEC counterpart, although further studies are necessary to clarify this point. NSMP NEEC includes both pure and mixed NEEC, mostly of large cell type, and seem associated with poor prognosis. Pure small cell NEEC, similarly to its pulmonary counterpart, appears to be p53abn and have poor prognosis [69].

## 9. Mesonephric-Like Carcinoma (MLEC)

MLEC is a rare entity which shows morphological and immunohistochemical features overlapping with cervical mesonephric carcinoma. MLEC may show different growth patterns, such as tubular, glandular, retiform, sex cord, papillary, hobnail, glomeruloid, and sieve-like, and may easily be misdiagnosed as other histotypes; solid and spindle-cell areas reflect poor differentiation and may show altered immunophenotype. The typical immunophenotype includes positivity for the “mesonephric markers” (i.e., luminal CD10 expression, GATA3 and TTF1 expression), negativity (or weak/focal positivity) for estrogen and progesterone receptor, patchy p16 positivity, and p53-wild-type expression pattern [4,70].

At a molecular level, MLEC is associated with KRAS mutations. Unlike mesonephric carcinomas, MLEC may show molecular alterations typical of endometrioid carcinoma, such as PTEN and CTNNB1 mutations, indicating a possible Müllerian origin with a secondary acquisition of a Wolffian phenotype [71]. The few available data about the TCGA groups suggest that MLEC is not associated with MMRd or POLEmut signatures [72]; the p53-wild-type pattern supports the absence of a p53mut signature as well. Therefore, it seems that MLEC falls into the NSMP group. Since the biological behavior of MLEC is consistently described as aggressive [72,73], the inclusion of this entity in the high-risk non-endometrioid group appears justified.

## 10. Gastric-Type Carcinoma (GTEC)

GTEC is an uncommon mucinous neoplasm which resembles endocervical gastric-type adenocarcinoma. GTEC has also been termed “gastrointestinal-type” carcinoma, given the spectrum of gastric and intestinal differentiation that may be found in these lesions [74]. While mucinous carcinoma is now considered as a type of differentiation of endometrioid carcinoma, GTEC is considered as a separate entity [4]. GTEC seems to be associated with gastric-type mucinous metaplasia of endometrium, which might be its precursor lesion. Diagnostic criteria proposed for GTEC include (i) voluminous, pale eosinophilic or clear cytoplasm with distinct cell borders and/or goblet cells, in the absence of other primary sites (ii) positivity for at least focal for one or more gastrointestinal markers (CK20, CDX2, MUC6), (iii) absence of an endometrioid component, (iv) estrogen receptor expression <5%, and (v) exclusion of cervical involvement [74]. However, recent reports showed a wide morphological heterogeneity in GTEC, which may affect its identification [75,76]. To date, there are still no data regarding the TCGA groups in endometrial GTEC. In endocervical GTEC, genomic profiling showed frequent (about 40%) TP53 mutation, although with low level of somatic copy number alterations and occasional microsatellite instability in the absence of high mutational burden [77]. If endometrial GTEC is similar to its endocervical counterpart, it is possible that it does not fit to the TCGA group of endometrial carcinoma. It is important to note that endometrial carcinomas with mucinous features have traditionally been considered as type I carcinomas [36]; instead, GTEC appears to be an aggressive entity, often presenting with adnexal involvement, and seems not to be estrogen-dependent (as demonstrated by the absence of hormone receptors expression) [74]. Therefore, it appears appropriate to consider GTEC together with non-endometrioid carcinomas in terms of patient management. Regarding the cases of EEC with gastrointestinal differentiation, a recent review suggested that they might be more aggressive than pure EC and associated with a MMRd signature [78]. Further studies are warranted in this regard.

## 11. Discussion

### 11.1. Prognostic Consistency of Molecular Groups

The emphasis linked to the TCGA molecular groups has led to the hypothesis that molecular data might completely replace the traditional histopathological assessment [79]. However, the four TCGA groups may vary with regard to clinical and pathological features [22,80]; these factors may have a different prognostic impact across the several groups. For instance, the POLEmut group might be the least affected by other clinicopathological features. Indeed, the favorable prognostic value of the POLEmut group appears not to significantly change when normalized for possible confounding factors [13]. The excellent prognosis of the POLEmut group is maintained across the several histotypes, justifying the guidance of the ESGO/ESTRO/ESP guidelines [12,23,33,34,35,43,44,52,63,64]. On the other hand, the prognosis of the NSMP group appears heavily affected by tumor grade and histotype. Indeed, the NSMP group shows a prognosis good-to-intermediate in low-grade EEC, intermediate in high-grade EEC, and poor in non-endometrioid carcinomas [12,23,33,34,43,44,52,63,64]. Furthermore, as discussed for EEC, the NSMP group may further be stratified based on histological (i.e., LVSI, depth of myometrial invasion), immunohistochemical (i.e., L1CAM expression), or molecular (i.e., CTNNB1 mutation) features [17]. Consistently with the lack of molecular signatures, the NSMP, therefore, lacks a univocal prognostic significance. The p53abn group consistently showed a poor prognosis in all histotypes, justifying the classification of all p53abn carcinomas in the high-risk group [12,23,33,34,35,43,44,52,63,64]. Prognostic differences between different histotypes may still exist, since the prognosis of this group can be further worsened by unfavorable clinicopathological factors [13]. For instance, p53abn SC might be more aggressive than p53abn EC but less aggressive than p53abn [23,60,61,62]. However, it is difficult to define if such differences may justify a different management. Regarding the MMRd group, its prognosis seems to consistently remains intermediate across different subsets of endometrial carcinomas. In early-stage, low-grade ECs, which usually have a good prognosis, MMRd appears as a risk factor for recurrence [81]. In high-grade ECs, which are characterized by a prognosis intermediate between low-grade EC and non-endometrioid carcinomas, MMRd is associated with intermediate prognosis [23,34]. In non-endometrioid carcinomas, which have a poor prognosis, MMRd is a favorable prognostic factor [35,43,44,63,64]. This suggests the possibility of considering the MMRd group as an intermediate-risk group regardless of the histotype. An exception would be UEC/DEC, in which a loss of SWI/SNF proteins expression appears associated with aggressive behavior even in the case of an MMRd signature [52,82].

### 11.2. Interpretation Issues and Multiple Classifiers

The correct assignment of endometrial carcinomas to one of the molecular subgroups requires a correct interpretation of molecular and immunohistochemical data. For instance, not all POLE mutations impact on prognosis. A large majority of POLE mutations outside the exonuclease domain are not associated with a ultramutated phenotype, and part of the mutations inside the exonuclease domain are not pathogenetic [83]. Recognizing the pathogenetic mutations of POLE is, therefore, necessary to classify a carcinoma as POLEmut. The identification of a p53abn signature requires a correct interpretation of p53 immunohistochemistry to predict the presence of TP53 mutations [84]. Most cases of TP53-mutant carcinomas show immunohistochemical overexpression of p53, defined as a diffuse and uniformly strong nuclear expression of p53 in >80% of tumor cells. Endometrial carcinomas with nuclear p53 expression in <80% of cells and/or with variable intensity should not be interpreted as p53abn. Other mutation-type patterns include a complete absence of p53 expression (in the presence of a positive internal control) and an unequivocal cytoplasmic expression. Additionally, a wild-type pattern could be observed in presence of p53 truncating mutation. Moreover, we have to keep in mind that some G3 EEC or ambiguous carcinomas with a mutator phenotype (either POLEmut or MMRd) can acquire a TP53 mutation later in the tumoral course, developing a subclonal TP53 mutation that may result in heterogenous p53 expression, with a combination of normal wild-type and abnormal patterns (overexpression and/or ‘null’ phenotype and/or cytoplasmic staining). In these cases, an experienced gynecopathologist together with an optimized immunohistochemical protocol are necessary to assess such patterns [85], distinguishing them from the possibility of mixed cancers, wild type variability and p53 mosaic patterns. For MMR immunohistochemistry, “deficiency” has usually been defined as a complete loss of expression in tumor cells with an internal positive control (stromal cells) [86]. A loss of MLH1 is typically accompanied by a loss of PMS2, while a loss of MSH2 is accompanied by a loss of MSH6. Losses of MSH6 or PMS2 may occur alone instead. On this account, it has been suggested that testing only MSH6 and PMS2 may have the same accuracy as the full MMR panel, with half the cost [87]. It should be remarked that MMR immunohistochemistry is very fixation-sensitive: poorly fixed areas typically show negative staining in the absence of stromal staining, with a gradual decrease of intensity from positive areas. Furthermore, some MMRd cases may show very weak and focal MMR expression in tumor cells, in the presence of an unequivocal positivity in stromal cells [86]. Finally, MMR loss may be subclonal; in these cases, a loss of expression in at least 10% of the tumoral area has been suggested as a cutoff to assign the tumor to the MMRd group [88]. Loss of both MSH2 and MSH6 suggests a mutation in MSH2; loss of both MLH1 and PMS2 suggests an underlying mutation or methylation in MLH1 and loss of MLH1 alone typically suggests underlying methylation. There is also a great variety of ambiguous staining patterns that should be considered:

- Geographical loss of MLH1 and PMS2 due to heterogeneous hypermethylation within the tumor.
- Geographical loss of MSH6 and/or MSH2 due to a secondary (non-germline) mutation in an MSH6 coding mononucleotide tract or a mutation in POLE.
- Weak focal/patchy immunoreactivity for MSH6 can be seen with MSH2 loss of expression/germline mutations.
- Subclonal loss of MMR protein expression.
- MSS/MSI-Low with loss of MMR protein expression due to MLH1 promoter hypermethylation or somatic MMR variants.
- MSI with retained/proficient MMR protein expression due to POLE variants.

In the case of simultaneous presence (3% of endometrial cancers) of two or three molecular signatures (the so-called “multiple classifier”: MMRd/p53abn; POLEmut/p53abn; MMRd/POLEmut/p53abn; MMRd/POLEmut), it should be remembered that outcomes correspond to those predicted by the driver molecular subtype; in particular, the POLEmut signature, when characterized by a pathogenic status, prevails over the other signatures, conferring a good prognosis regardless of MMR and p53 status, while the MMRd signature prevails over the p53abn signature [89].

### 11.3. Specific Treatment Options

We extensively discussed the impact of molecular groups on the risk stratification and management of endometrial carcinoma. In addition, molecular signatures may predict susceptibility of endometrial carcinoma to specific therapeutic agents. Given their high mutational load, abundance in activated cytotoxic TILs and peritumoral CD8+T-cells, and a greater density of neoantigens, the POLEmut and MMRd groups, defined hot tumors, may benefit from immunotherapy [90]. In particular, they are considered optimal candidates to respond to anti-PD-1/PDL1 treatment (immune checkpoint blockade therapy). Assessing the expression of PDL-1 may be reasonable in these tumors. Different scoring algorithms, by the use of different immunohistochemical commercially available antibody clones and different cut-offs, have been proposed, evaluating PD-L1 positivity in tumor cells (TCs) and/or in immune cells (ICs) separately or in combination (combined positive score, CPS) [90,91]. Regarding endometrial carcinoma, the scientific literature reported considerable variations in PD-L1 positivity frequencies (from 0.9% to 44.3%) and evidenced different PD-L1 expression profiles between molecular subclasses, histologic subtypes, and tumoral stage, with the POLE mutant, the MMR deficient, the non-endometrioid types, and the advanced endometrial cancers displaying the highest PD-L1 levels in TCs and ICs, and with the highest CPS. In particular, it seems that CPS have methodological advantages over cell type-specific scoring systems [91]. Finally, the recent ESGO/ESTRO/ESP guidelines of endometrial carcinoma have approved MMRd/microsatellite instability as the selection criteria for second line anti-PD-1/PD-L1 based immune therapy with pembrolizumab. A combined therapy (Pembrolizumab+Lenvatinib, a multi-tyrosine kinase inhibitor) could be considered an option as second line therapy in MMR stable ECs. In the p53abn group, HER-2 amplification has been described in a subset of SEC [92]; anti-HER-2 targeted therapy with trastuzumab has shown promising results in these tumors [93]. Since HER-2 overexpression has also been described in UCS (which is p53abn in most cases) and in p53abn CCEC, it is possible that these tumors may be sensitive to anti-HER-2 targeted therapy [94,95]. Furthermore, a subset of p53abn carcinomas shows high DNA damage and high PARP-1 expression, offering the possibility of using PARP-inhibitors to treat these cases [96].

### 11.4. CTNNB1-Mutant: The Fifth Molecular Group?

Several studies suggested that low-grade, early-stage EECs harboring CTNNB1 exon 3 mutations have worse OS and recurrence-free survival, as also described for MMR deficiency [81,97,98], although their frequent clinic pathological characteristics are commonly associated with lower relapse risk (younger age, squamous differentiation, low TILs, less incidence of deep myometrial invasion, and less incidence of LVSI, with a low number of other concurrent mutations, such as KRAS and FGFR2 mutation). As discussed above, the Leiden/PORTEC group showed that the CTNNB1 exon 3 mutations had an independent prognostic value within the NSMP group, identifying a subset at intermediate prognosis (similar to that of the MMRd group) [17]. On this account, it has been proposed that CTNNB1-mutant cases might constitute the fifth molecular group of endometrial carcinoma [79,99]. Since CTNNB1 mutations are found in about half of NSMP EECs, the CTNNB1-mutant group would account for about 20% of all endometrial carcinomas [12]. It is necessary to remark that the CTNNB1-mutant signature would have a prognostic value only in the absence of other molecular signatures (POLEmut, MMRd, or p53abn), and reasonably only in EECs. Furthermore, the Leiden/PORTEC group only included EECs classified as “high-intermediate-risk” cases; this highlights the need for further studies to generalize the prognostic role of CTNNB1 [17]. Since CTNNB1 mutations may be associated with nuclear accumulation of β-catenin, immunohistochemistry has been assessed as a possible surrogate for sequencing to identify CTNNB1-mutant cases. The results support an overall good agreement between β-catenin expression and CTNNB1 status [98,99,100]. However, it seems that nuclear accumulation of β-catenin in EEC implies the presence of CTNNB1 mutation, but not vice versa [100,101]. Furthermore, there are no defined criteria to classify β-catenin expression as aberrant; in fact, even the presence of few positive nuclei seems to be associated with CTNNB1 mutation, while no clear data are provided regarding the intensity of staining [100]. Finally, in most cases, nuclear β-catenin accumulation in EEC is limited to the areas of morular metaplasia, which is not present in all CTNNB1-mutant cases and does not seem to be associated with prognosis [100,101,102,103]. Therefore, there is still insufficient evidence to introduce the use of β-catenin immunohistochemistry in the prognostic stratification of endometrial carcinoma.

### 11.5. Unsolved Problems: Preoperative Setting and Interlaboratory Reproducibility

Nowadays, an intriguing challenge is represented by the application and validation of TCGA classification in small diagnostic biopsies/endometrial curetting, in order to decide the surgical approach, since high grade POLE mutated tumors may benefit from reduced extent of surgery. Recent studies suggest highly concordant results in diagnostic biopsies and hysterectomy specimens, in particular for MMR loss, MSI high, p53 wild, and aberrant types, in contrast to moderate levels of agreement reported for the classical histomorphological parameters (grade, histotype). In this way, molecular classification preoperatively applied seems to provide earlier and more reliable prognostic information to guide clinical management [104]. The bioptical specimen also allow a better tissue fixation and a superior antigen preservation, consequently ensuring a more reproducible and adequate biological characterization [105]. Finally, intratumoral heterogeneity may be a challenge for adoption of the molecular TGCA classification to small biopsies in daily practice.

## 12. Conclusions

The integration of the TCGA molecular groups in the ESGO/ESTRO/ESP guidelines of endometrial carcinoma have offered the possibility of limiting the current under- and overtreatment of women with endometrial carcinoma. However, there are still several limitations that should be overcome, especially regarding the management of MMRd and NSMP carcinomas. Major improvements that might be hypothesized are (i) lumping together MMRd carcinomas, regardless of grade and histotype (with the exception of SWI/SNF-deficient UEC/DEC) and (ii) substratifying the risk in NSMP EECs based on further prognostic factors (e.g., CTNNB1 mutations). Moreover, methods and criteria to apply the molecular classifier are not without pitfalls and limitations and are expected to evolve over time. Further improvements in the prognostic definition of endometrial carcinomas will be achieved only integrating clinical, histomorphological, immunohistochemical, and molecular data in a multidisciplinary approach.

## Figures and Tables

**Table 1:** Distribution of risk categories according to stage and histology.

LOW RISK	Stage I-II POLEmut Stage IA MMRd/NSMP low-grade EEC with no or focal LVSI
INTERMEDIATE RISK	Stage IB MMRd/NSMP low-grade EEC with no or focal LVSI Stage IA MMRd/NSMP high-grade EEC with no or focal LVSI Stage IA p53abn EEC with no myometrial invasion Stage IA non-endometrioid with no myometrial invasion
HIGH-INTERMEDIATE RISK	Stage I MMRd/NSMP EEC with LVSI Sage IB MMRd/NSMP high-grade EEC Stage II MMRd/NSMP EEC
HIGH RISK	Stage III–IVa EEC with no residual disease Stage I–IVa p53abn EEC with myometrial invasion and no residual disease Stage I–IVa non-endometrioid * with myometrial invasion and no residual disease
ADVANCED/METASTATIC	Stage III–Iva with residual disease Stage IVb

* MMRd and NSMP clear cell carcinomas are not included in any risk category because their biological behavior is undefined.

## Abbreviations

TCGA	The Cancer Genome Atlas
POLE	DNA polymerase epsilon
MSI	microsatellite instability
ESGO/ESTRO/ESP	European Society of Gynaecological Oncology, European SocieTy for Radiotherapy and Oncology, European Society of Pathology
USA	United States of America
EEC	endometrial endometrioid carcinoma
SEC	serous endometrial carcinoma
CCEC	clear cell endometrial carcinoma
UEC/DEC	Undifferentiated/dedifferentiated endometrial carcinoma
UCS	uterine carcinosarcoma
NEEC	neuroendocrine endometrial carcinoma
MLEC	mesonephric-like endometrial carcinoma
GTEC	gastric/gastrointestinal-type endometrial carcinoma
LVSI	lymphovascular space invasion
FIGO	International Federation of Gynecology and Obstetrics
DNA	Deoxyribonucleic Acid
SCNA	somatic copy-number alteration
MMR	mismatch repair
MMRd	MMR-deficient
p53abn	p53-abnormal
NSMP	no specific molecular profile
AEH/EIN	atypical endometrial hyperplasia/endometrioid intraepithelial neoplasia
MELF	microcystic, elongated, and fragmented
NCCN	National Comprehensive Cancer Network
TCs	Tumor cells
ICs	immune cells
CPS	combined positive score
